# Disease severity, debridement approach and timing of drug modify outcomes of adjunctive azithromycin in non-surgical management of chronic periodontitis: a multivariate meta-analysis

**DOI:** 10.1186/s12903-019-0754-0

**Published:** 2019-04-27

**Authors:** Nithya Jagannathan, Aneesha Acharya, Ong Yi Farn, Kar Yan Li, Luigi Nibali, George Pelekos

**Affiliations:** 1 Periodontology, Faculty of Dentistry, The University of Hong Kong, 3F, The Prince Philip Dental Hospital, 34 Hospital Road, Sai Yin Pun, Hong Kong; 2 Dr D Y Patil Dental College and Hospital, Pimpri, Pune, India; 3 Centre for Oral Immunobiology & Regenerative Medicine, Centre for Oral Clinical Research, Institute of Dentistry, Barts and the London School of Medicine and Dentistry, Queen Mary University London, London, England

**Keywords:** Azithromycin, Periodontitis, Non-surgical periodontal therapy, Meta-analysis

## Abstract

**Background:**

Past meta-analyses have shown adjunctive systemic Azithromycin (AZI) to provide minor clinical benefits in scaling and root surface debridement (S/RSD). However, these have not considered the covariance of key outcome parameters; probing pocket depth (PPD) and Clinical Attachment Level (CAL) or systematically examined some potential sources of heterogeneity.

**Aim:**

To jointly synthesize 6-month outcomes of systemic AZI as adjunctive to S/RSD in chronic periodontitis and investigate 3 potential sources of heterogeneity.

**Methods:**

Four databases were searched for suitable randomized controlled clinical trials (RCTs). Standardized mean differences (SMD) in PPD and CAL between AZI + S/RSD and S/RSD alone, at 6-month follow-up were computed. Within-study covariances of PPD and CAL were derived from reported multiple time-point data. A multivariate meta-analysis with random effects jointly modelled PPD and CAL, factoring in their covariance. This model included 3 moderators with interaction effects; timing of AZI initiation (pre-therapy/post-therapy), type of S/RSD [full-mouth debridement (FMD)/partial-mouth debridement (PMD)], and baseline study-level mean values of PPD/CAL.

**Results:**

Among 276 abstracts, 11 observations from 9 RCTs qualified for meta-analysis. Within-study correlation-coefficients of PPD with CAL significantly increased with increasing study-level baseline mean values (Spearman’s r = 0.79, *p* < 0.01). The full multivariate meta-analysis model showed significant effects for the 3 moderators (Q statistic = 150.03, *p* < 0.01), retained significant residual heterogeneity (Q statistic = 88.50, p < 0.01) but outperformed (Likelihood- ratio statistic = 102.95, p < 0.01,) a null-model with no moderators (Q statistic = 201.5, *p* < 0.01). A significant effect was seen only on the SMD for PPD (estimate = 1.16 mm, 95% CI: 0.27 mm–2.07 mm mm, *p* = 0.01) but not CAL (estimate = 0.17 mm, 95% CI: -0.92 mm-1.26 mm, *p* = 0.76). SMD in PPD positively interacted with study baseline value (estimate = 0.11, 95% CI: 0.08–0.15, *p* < 0.01). Significant negative interactions of SMD in PPD with PMD (estimate = − 1.25 mm, 95% CI: -1.73 mm- -0.78 mm, p < 0.01) and pre-therapy drug initiation (estimate = − 1.18 mm, 95% CI: -1.48 mm--0.87 mm, *p* < 0.01) were evident.

**Conclusion:**

Joint synthesis of PPD and CAL showed, at 6-months, AZI + S/RSD provided a benefit over S/RSD alone for PPD alone when correlation with CAL was accounted for. Deeper study-level baseline PPD, FMD type of S/RSD, and post-therapy drug initiation associated with greater PPD reduction.

**Electronic supplementary material:**

The online version of this article (10.1186/s12903-019-0754-0) contains supplementary material, which is available to authorized users.

## Introduction

Clinical benefits of antibiotics as adjuncts to scaling and root surface debridement (S/RSD) are recognized, although overall small in magnitude [1–3]. Factors that could explain heterogeneous outcomes in this regard are not systematically examined. An understanding of such factors is critical to enable selection of the right patient, where the clinical benefit of adjunctive antibiotics outweighs risks of antimicrobial resistance, a key issue concerning antimicrobial use. Furthermore, as initial benefits of antibiotics as adjuncts to S/RSD may not be sustained after a single course [4, 5], there is a need for evidence synthesis at long post-therapeutic periods. Among antimicrobial agents, Azithromycin (AZI) is concentrated in fibroblasts, phagocytes, [6] and gingival tissues [7] and possesses anti-inflammatory, immunomodulatory characteristics [8]. As compared to the most documented adjunctive antimicrobial regimen of Amoxicillin and Metronidazole, systemic AZI entails higher patient compliance, a longer therapeutic window, and fewer side effects [9, 10]. Four previous systematic reviews have summarised the evidence regarding AZI as an adjunct to S/RSD [11–14]. Two reviews did not perform meta-analyses, citing high trial heterogeneity [11, 12]. The two existing meta-analyses have analyzed changes in probing pocket depth (PPD) and clinical attachment level (CAL) in separate analyses [13, 14]. In clinical populations, these two outcomes can be significantly correlated or inter-dependent. The strength of such correlation, however, is shown to vary widely, depending upon the disease severity [15]. As a result, the effect of different within-study correlations of these two key outcomes can result in bias if ignored [16, 17]. A multivariate meta-analysis can simultaneously analyze multiple correlated outcomes [18]. This approach has several advantages over univariate approaches. It generates improved estimates of effect sizes of correlated outcomes and estimates their association, which may be useful for prediction [16, 17]. Recognizing this fact, a multivariate meta-analysis approach has been successfully applied to the primary periodontal parameters PPD and CAL [19]. A similar approach would be valuable to estimate the true effects of systemic AZI as an adjunct to S/RSD.

Other aspects of data synthesis in the two existing meta-analyses suggest directions for further research. One meta-analysis summarised evidence from trials of both systemic and local AZI in chronic or aggressive periodontitis [13], which could underpin variability in outcomes. Here, the outcome measures were pooled estimates of 12improvements in clinical parameters in treatment and control groups each. A large effect for each group was noted. However, as there was no statistical comparison of these effects a clear conclusion regarding their comparative benefits remained unclear [13]. A second meta-analysis that summarised evidence from randomized clinical trials of adjunctive systemic AZI to S/RSD in chronic periodontitis alone used raw mean differences in clinical parameters between treatment and control group as the outcome [14]. A limitation is that differences in numbers of subjects per study is not accounted for in this approach, and may be a source of bias. This study analyzed 2 potential sources of heterogeneity by subgroup analyses; the severity of disease and the post-treatment observation time. Additional factors could drive variability in the clinical outcomes of adjunctive AZI. S/RSD may be delivered as a full-mouth debridement (FMD) instead of the typical partial mouth debridement (PMD) regime such as a quadrant-wise approach [20]. FMD may itself confer additional treatment benefit [20, 21]. The timing of drug initiation, either preceding biofilm removal or afterward, varies across trials as there is no widely accepted standard and may also impact outcomes [22]. The multivariate approach can jointly synthesize correlated outcomes while describing the effects of potential moderators. In addition, the between-study random variance may be estimated in this advanced meta-analysis approach. Therefore, the current study aimed to; i) jointly synthesize the benefits in key periodontal outcomes (PPD and CAL) due to systemic AZI as an adjunct to S/RSD and, ii) investigate 3 potential moderating factors, by applying a multivariate meta-analysis approach.

## Methods

A detailed protocol was designed based on the PRISMA guidelines [23] and the study was conducted following recommendations from the Cochrane Collaboration [24]. The study protocol was registered in the PROSPERO database (PROSPERO 2018, ID = CRD42018093238).

### Focused question

The focused question addressed in this study is “What is the clinical benefit of systemic azithromycin as an adjunct to scaling and root debridement in the treatment of chronic periodontitis as compared to scaling and root debridement alone at 6 months post-therapy and how do 3 selected factors (timing of AZI initiation, type of S/RSD and baseline study-level mean values of PPD/CAL) modify its outcomes?”

### Search methods

Pubmed (https://www.ncbi.nlm.nih.gov/pubmed/), Embase (http://www.embase.com/home) and Cochrane library (http://www.cochrane.org) were initially searched for papers published up to February 2018, to answer the focused question. A broad search strategy was employed to capture as many relevant studies as possible. The following keywords were used: “Azithromycin”, “Periodontitis”, “Periodontal Diseases”, “Periodontal Treatment” and “Periodontal Therapy”. MeSH terms were used when the search engine of the database permitted. OpenGrey database was searched for unpublished reports (http://www.opengrey.eu/). The search strategy for Pubmed is presented in Additional file 1: Table S1. A manual search included issues in the past 20 years of the following journals: Journal of Periodontology, Journal of Clinical Periodontology, Journal of Dental Research, Journal of Dentistry and Journal of the American Dental Association. The reference lists of articles eventually included were also searched, to identify additional relevant studies until no new applicable titles appeared (saturation).

### Study selection

In the first phase, two blinded reviewers (AA and GP) independently selected references based on titles and abstracts. From within this initial selection, studies were further admitted into the second phase where the full-text analysis was conducted based on the following predetermined inclusion criteria:

1. Randomized controlled clinical trial.
2. Patients diagnosed with Chronic Periodontitis.
3. Studies comparing S/RSD (defined as ‘scaling and root planing’)+AZI versus S/RSD.
4. Studies conducted on systemically healthy adult patients.
5. Follow-up of at least 6 months reporting data on clinical parameters (CAL, PPD, BOP) at the 6-month time-point.
6. Publication in English.

These included studies were then qualitatively assessed. The next phase involved quantitative analysis. For this purpose, studies that described changes in periodontal outcomes and associated standard deviation (SD) (or data used to calculate them) were included. Displayed data which could not estimate these outcomes were excluded. The agreement between reviewers was determined by the κ statistics and any disagreements were resolved by discussion until a consensus was reached.

### Assessment of risk of bias

The two independent reviewers evaluated the methodology of the included papers. (OYF and GP). Studies were appraised based on the Cochrane Risk of Bias Tool 25 and the recommendations of the CONSORT statement [25]. Seven domains were assessed and judged as either at low, unclear or high risk of bias (1.Selection Bias\2.Allocation Bias \3.Performance Bias \4.Detection Bias \5.Attrition Bias \6.Reporting Bias \7.Inclusion/Exclusion Criteria.) A low risk of bias (L) was assigned when all these criteria were met. When one or two of the criteria were not met a moderate risk of bias (M) was assigned and when 3 or more were violated, a high risk of bias (H) was considered. Furthermore, a summary assessment of the risk of bias of all outcomes across domains and across studies was conducted.

### Outcome measures

The primary outcome measured changes in Probing Pocket Depth (PPD) and Clinical Attachment Levels (CAL).

### Data extraction and management

Data were collected by the primary reviewer (AA) and systematically inserted into a table, using Microsoft Excel. Duplicate data extraction was done by the second reviewer (NJ) and the results were merged. Data on demographic characteristics, study design, intervention methodology and dosage, periodontal maintenance, follow up period and adverse effects were extracted from included studies. Numerical data was extracted from the presented tables and standard errors were converted to standard deviation (SD). Extracted data were cross-checked three times and any disagreements were resolved by discussion.

### Statistical analysis

The data extracted from the selected studies were entered manually into a database. Mean values, standard deviations, and numbers of treated subjects in test and control groups were extracted. If no standard deviation was available then it was calculated based on standard error and sample size, in accordance with the Cochrane Handbook for Systematic Review of interventions and used as the outcome measure in the meta-analysis. Within-study correlation measures of PPD and CAL were estimated from the summary statistics of the repeated PPD and CAL measures reported for multiple times points (3–6 time points were reported per study). These correlation values were then used to compute covariance and generate a variance-covariance matrix. The final meta-analysis model was fit using standardized mean difference (SMD) as the effect size [26] using a restricted maximal likelihood (REML) estimator, using the R package ‘*metafor’* with the function ‘rma.mv’ (https://cran.r-project.org/web/packages/metafor/). The study was used as a random variance component. The full model included 3 moderators; timing of drug, type of S/RSD, and Baseline study mean value of outcomes (CAL, PPD). The interactions of each with the outcome variables were modeled. Model heterogeneity was assessed by the Q statistic and test and an omnibus test assessed moderator significance. A reduced model without any moderators but only the study-level random variable was also computed and the full model and the reduced model were compared using log-likelihood ratio tests (LRT) and funnel plots for visualization of heterogeneity.

## Results

### Study selection

**Fig. 1:**
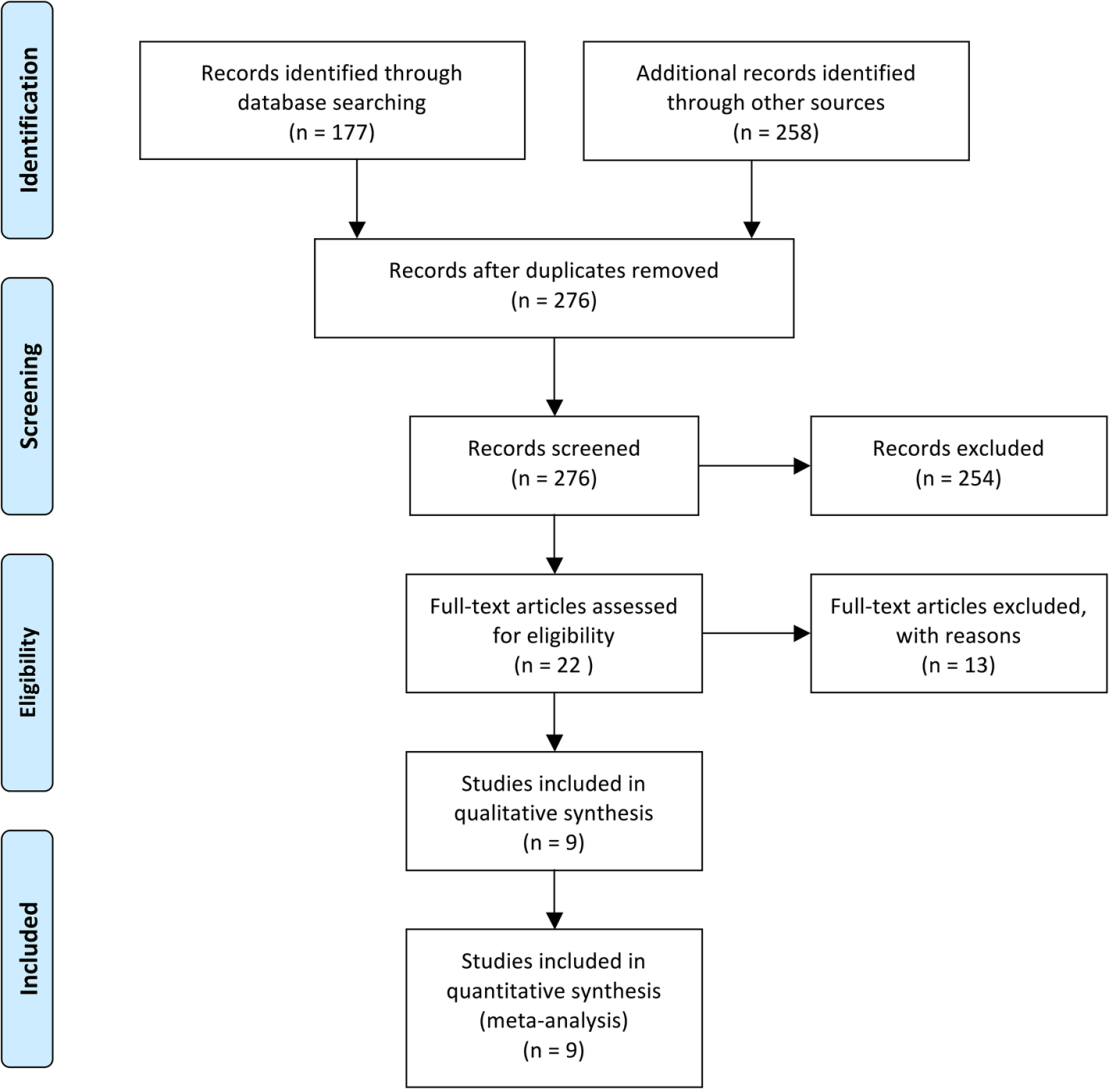
PRISMA flowchart showing the selection of studies

The electronic and manual search yielded a total of 276 abstracts. During the first step of the study selection process, 254 publications were excluded based on the evaluation of titles and abstracts (κ = 0.9327). In the second phase, complete full-texts of the remaining 22 studies were thoroughly evaluated. A total of 13 articles were excluded in this phase because they did not fulfill the inclusion criteria (κ =1.000) (Additional file 1: Table S2). The remaining 9 articles were selected for qualitative analysis. Nine of these were included in the meta-analysis for PPD and CAL, and 8 were included for BOP. A flowchart of the study selection process is shown in Fig. 1.

### Quality assessment

**Fig. 2:**
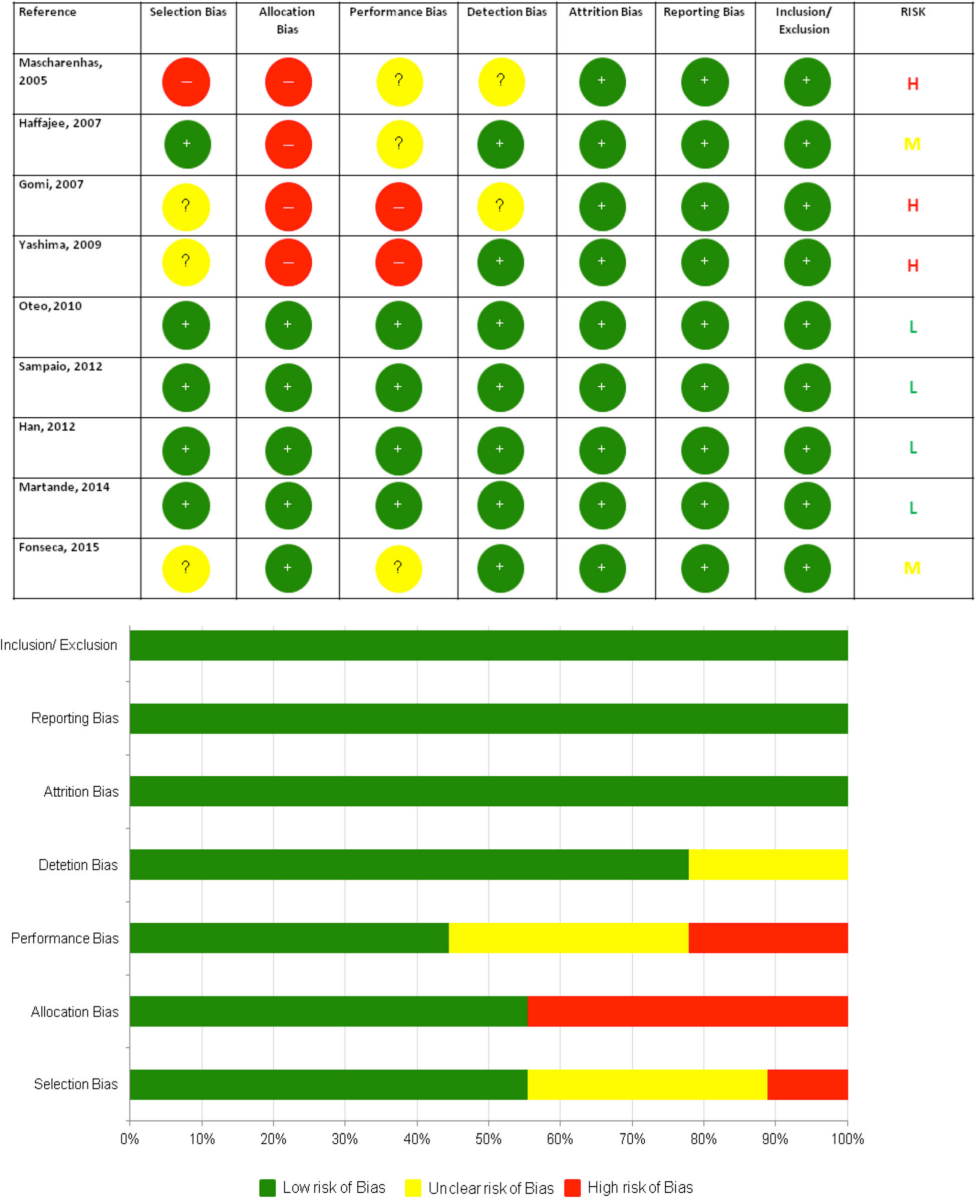
Summary of quality assessment of included studies

Quality assessment of the 9 RCTs revealed that 4 studies were at low risk of bias. Three studies were at high risk of bias since they failed to detail the randomization process, masking methodology, and sample size calculation which estimates the minimum number of participants required to detect a difference among the groups [25, 27, 28]. A summary of the quality assessment is presented in Fig. 2. High risk of selection bias was reported in 11% of the included trials due to non-random approaches in categorizing the participants. Allocation bias was observed in 55.5% of the trials due to inadequate concealment of the enrolling participants. Performance Bias was reported in 22% of the included trials as the participants and investigator were incompletely blinded during the study.

### Summary of studies (based on the PICO guideline): population

Clinical trials comprised a total of 393 participants with 211 males, with a minimum of 28 to a maximum of 164 patients with mean age spanning from 32 to 51. The study centers were from a varied population of different ethnic backgrounds. Table 1 summarizes the main characteristics of the included studies.

**Table 1:** Characteristics of the included studies

Author/Year	n/ Country	n (Female/Male)	Age (years)	Project Design	Intervention Methodology	Nature of S/RSD	Intervention Groups	Follow-Up	Periodontal Maintenance	Adverse Events
Mascarenhas et al. 2005 [28]	30/ USA	AZI: 15 (4/11) C: 15 (7/8)	AZI: 47.0 ± 10.1 C: 45.3 ± 10.8	Parallel, Single Blinded	AZI: Post therapy, End of S/RSD, in 2 sessions within 14 days C: S/RSD in 2 sessions within 14 days.	Qu S/RSD(2 sessions)	AZI: 500 g × 1 day + 250 g × 4 days C: S/RSD	3, 6 months from baseline	3rd, 6th months by examiner	Not reported
Gomi et al. 2007 [25]	34/ Japan	AZI: 17 (9/8) C: 17 (9/8)	AZI: 45.4 ± 14.3, C: 51.0 ± 8.8,	not described	AZI: Pre-therapy, 3 days prior to S/RSD C: S/RSD in 4–6 times, weekly intervals	FMD	AZI: 500 g × 3 days C: S/RSD	1, 3, 6 months from baseline	Weekly by Dental Hygienist	Diarrhoea (*n* = 1)
Haffajee et al. 2007 [32]	48/ USA	AZI: 25 (10/15), C: 23 (7/16)	AZI:47 ± 14, C:43 ± 15	Parallel. Single Blinded	AZI: Pretherapy, Start of Quadrant Wise S/RSD C: S/RSD in 4 weekly intervals	Qu S/RSD (4 sessions)	AZI: 500 g × 3 days C: S/RSD	1, 3, 6, 12 months from baseline	1st, 3rd, 6th and 12th months	Difficulty in swallowing (n = 1) Allergic reaction (*n* = 1)
Yashima et al. 2009 [27]	30/ Japan	AZI 1: 10 (6/4), AZI 2: 10 (5/5), C: 10 (4/6)	AZI 1: 51.1 ± 11.6, AZI 2: 50.8 ± 14.2, C: 51.0 ± 10.6	not described	AZI 1: Pre therapy 3 days prior to S/RSD (Full Mouth) AZI 2: Pre therapy 3 days prior to S/RSD. Conducted in 3 times, within 7 days C: S/RSD in 6 sessions with 1 week interval	FMD Qu S/RSD (3 sessions)	AZI 1: 500 g × 3 days AZI 2: 500 g × 3 days C: S/RSD	1, 3, 6, 9, 12 months from baseline	Once a month by the Dental Hygienist	Diarrhoea (n = 1)
Oteo et al. 2010 [30]	28/ Spain	AZI: 15 (8/7), C: 13 (5/8)	AZM: 46.6, C: 7.1	Parallel, Double Blind, Placebo Controlled.	AZI: Post therapy, End of S/RSD in 2 times, within 7 days C: S/RSD in 2 times, within 7 days.	Qu S/RSD –(2 sessions)	AZI: 500 g × 3 days C: Placebo × 3 days	1, 3, 6 months from baseline	1st, 3rd, 6th months	Diarrhoea n = 1)
Sampaio et al. 2011 [34]	40/ Brazil	AZI: 20 (7/13), C: 20 (9/11)	AZI: 44.4 ± 7.42, C: 43.5 ± 5.90	Parallel, Double Blind, Placebo Controlled	AZI: Post therapy, End of S/RSD in 4–6 times, within 14 days C: S/RSD in 4–6 sessions within 14 days	Qu S/RSD (4–6 sessions)	AZI: 500 g × 5 days C: Placebo × 3 days	6, 12 months from baseline	Not reported	Diarrhoea (n = 1); Excessive Sleepiness (*n* = 3) Headache (n = 1)
Han et al. 2012 [33]	28/ Turkey	AZI: 14 (4/10), C: 14 (6/8)	AZI: 46.8 ± 5.1 C: 44.8 ± 5.0	Parallel, Double Blind, Placebo Controlled	AZI: Post therapy, End of quadrant by quadrant S/RSD in 4 sessions C: Quadrant by quadrant S/RSD in 4 sessions	Qu S/RSD (4 sessions)	AZI: 500 g × 3 days C: Placebo × 3 days	1, 3, 6 months from baseline	Not Reported	None
Fonseca et al. 2015 [29]	85/ Brazil	AZI 1: 15, C 1: 15 AZI 2: 14, C 2: 13	44.6 ± 8.8	Parallel, double blinded	AZI 1: Pretherapy, Start of full mouth S/RSD. C 1: Full mouth S/RSD within 24 h in 2 sessions. AZI 2: Pretherapy, Start of quadrant S/RSD. C 2: S/RSD per quadrant for 30 min in weekly interval between sessions	1. FMD (2 sessions in 24 h) 2. Qu S/RSD (4 weekly sessions)	AZI: 500 g × 3 days C: S/RSD alone	3, 6 months from baseline	Not reported	None
Martande et al. 2016 [31]	70/ India	AZI: 35 (14/21), C: 35 (16/19)	AZI: 33.3 ± 7.3 C: 32.6 ± 5.4	Parallel, Double Blind, Placebo Controlled	AZI: Post-therapy End of S/RSD in 3–5 times within 14 days. C: S/RSD in 3–5 times within 14 days	Qu S/RSD (3–5 sessions)	AZI: 500 g × 3 days C: Placebo × 3 days	1, 3, 6, 12 months from baseline	1st, 3rd, 6th, 12th week	

*AZI* Test group: Azithromycin + S/ RSD Group. *C* Control Group; SRSD alone, *FMD* Full mouth debridement, *PMD* Partial Mouth S/RSD,

(studies are ordered by year of publication)

### Intervention/comparison

Periodontitis was evaluated by charting up to 6 sites per tooth in all included studies. Follow-up periods varied from 6 to 12 months. Multiple time points (1–12 months) were reported for all 9 studies (Table 1), which enabled the estimation of study-level correlation between the PPD and CAL values. The time for prescription of AZI and the S/RSD approaches were variable. FMD in a single sitting was employed in 1 study [25], while 2 studies had subgroups which employed the use of both FMD and PMD performed over 3–4 sessions [27, 29]. The remaining six trials utilized a PMD approach with quadrant or sextant-wise S/RSD performed in 3–4 sessions at an average of 5–7 days interval [28, 30–34]. Regarding the prescription of AZI, 1 study administered 500 mg for 5 days [34]. Another study administered a 500 mg loading dose followed by 250 mg once daily from days 2 to 5 [28], while all other 7 studies used 500 mg once daily for 3 days. However, the time of drug initiation was variable. There were 5 studies which started the drug regime on the last day of treatment, whereas two studies started AZI, on the first day of treatment (Table 1). Both studies on Japanese cohorts initiated the drug prescription scheme 3 days prior to non-surgical treatment [25, 27]. In one study AZI was compared to other antibiotics, with the control group having only S/RSD [34]. Another trial compared the use of AZI and CHX mouth rinse each used as an adjunct to FMD type of S/RSD and to PMD type S/RSD performed quadrant-wise, with the control groups having S/RSD alone [29]. CHX treated groups were not considered in the present analysis.

### Outcomes; probing depths and clinical attachment levels

Study outcomes are summarized in Table 2. Seven studies documented an advantageous effect of systemically administered AZI compared to S/RSD alone with regards to PPD reduction. Significantly higher PPD change between 6 and 12 months for AZI adjunctive to S/RSD was reported in 3 studies, [27, 30, 32]. Four studies showed a beneficial effect of systemic AZI compared to S/RSD alone with regards to CAL gain [27, 30–32].

**Table 2:** Major clinical outcomes of included studies

Author/Year	Clinical Attachment Level	Probing depth
Mascarenhas et al. 2005 [28]	Baseline Study Mean: 3.87 mm CAL gain in shallow pocket, 6 month: Control - 0.11 mm; AZI - 0.55 mm (*p* < 0.05) CAL gain in moderate pockets, 6 month: Control − 1.52 mm (p < 0.05); AZI − 1.01 mm (p < 0.05). CAL gain in deep pockets, 6 months: Control - 1.32 mm (p < 0.05); AZI - 2.56 mm (p < 0.05), Between group difference: p < 0.05	Baseline Study Mean: 4.47 mm PPD reduction in shallow pocket, 6 month: Control: 0.02 mm; AZI: 0.43 mm (p < 0.05), PPD reduction in moderate pocket,, 6 month: Control: 1.0 mm (p < 0.05); AZI: 1.7 mm(p < 0.05). Between group difference: p < 0.05 PPD reduction in deep pocket,, 6 month: Control: 1.98 mm (p < 0.05); AZI: 3.52 mm (p < 0.05), Between group difference: p < 0.05
Gomi et al. 2007 [25]	Baseline Study Mean: 7.73 mm Control: Baseline: 7.21 ± 1.37 mm; 25 weeks: 5.74 ± 0.96 mm AZI: Baseline: 7.47 ± 1.96 mm; 25 weeks: 4.85 ± 1.05 mm. Between group difference: *P* > 0.05 at 13 and 25 weeks	Baseline Study Mean: 4.11 mm Control group: Baseline: 4.05 ± 0.68 mm; 25 weeks: 3.30 ± 0.36 mm AZI: Baseline: 3.98 ± 1.06 mm; 25 weeks: 2.36 ± 0.76 mm Between group difference: *P* < 0.001 at 13 and 25 weeks
Haffajee et al. 2007 [32]	Baseline Study Mean: 3.2 mm CAL gain: 6 months- Control:0.12 ± 0.15, AZI: 0.17 ± 0.12 (p < 0.05)	Baseline Study Mean: 3.01 mm PPD reduction: 6 months- Control: 0.45 ± 0.15 (p < 0.05), AZI: 0.103 ± 0.11 (*p* < 0.0001)
Yashima et al. 2009 [27] (FMD)	Baseline Study Mean: 3.99 mm Between FMD test control group difference: *P* < 0.01 at 1, 3, 6 months and *P* < 0.05 at 9 and 12 months Between PMD test control group difference: P < 0.01 at 1 month, P < 0.05 at 6, 9 and 12 months	Baseline Study Mean: 5.08 mm Between FMD test and control groups: P < 0.05 at 6, 9 and 12 months Between PMD test and control groups: P < 0.05 at 6, 9 and 12 months
Oteo et al. 2010 [30]	Baseline Study Mean: 3.51 mm CAL gain: 6 months- Control:0.29 mm(P > 0.05), AZI:0.76 mm(*P* = 0.004) Between group difference: *P* = 0.016 at 6 months	Baseline Study Mean: 2.92 mm PPD reduction: 6 month-Control: 0.34 mm (p < 0.05), AZI: 0.8 mm (p < 0.05) Between group difference: *P* = 0.009
Sampaio et al. 2011 [34]	Baseline Study Mean: 5.63 mm CAL gain at 1 year: Control: 2.35 ± 1.70, AZI: 2.68 ± 1.76, Between group difference P > 0.05	Baseline Study Mean: 4.92 mm PPD reduction at 1 year: Control: 3.83 ± 1.92, AZI: 3.45 ± 1.74 Between group difference P > 0.05
Han et al. 2012 [33]	Baseline Study Mean: 5.5 mm CAL gain in shallow pockets: 1 month-Control: 1.36 ± 0.5 mm; AZI: 1.47 ± 0.3 mm, 3 months-Control: 1.48 ± 0.6, AZI: 1.58 ± 0.4 mm, 6 months- Control:1.54 ± 0.5 mm, AZI: 1.55 ± 0.5 mm. CAL gain in moderate pockets: 1 month- Control: 0.33 ± 0.2 mm, AZI: 0.34 ± 0.2 mm, 3 months- Control: 0.31 ± 0.2 mm, AZI: 0.43 ± 0.3 mm 6 months-Control: 0.39 ± 0.4 mm, AZI: 0.34 ± 0.2 mm. CAL gain in deep pockets: 1 month- Control: 1.15 ± 2.3 mm; AZI: 1.99 ± 3.0 mm, 3 months- Control: 0.49 ± 0.7 mm, AZI: 1.25 ± 1.9 mm 6 months-Control: 0.54 ± 0.5 mm, AZI: 2.25 ± 3.1 mm. Oveall: P < 0.05 for Control and AZI both	Baseline Study Mean: 3.93 mm PPD reduction in shallow pockets: 1 month-Control: 1.44 ± 0.5 mm; AZI: 1.56 ± 0.4 mm, 3 months-Control: 1.54 ± 0.4 mm, AZI: 1.79 ± 0.4 mm, 6 months- Control: 4.45 ± 0.5 mm, AZI: 1.81 ± 0.5 mm PPD reduction in moderate pockets: 1 month- Control: 2.28 ± 0.3 mm, AZI: 2.18 ± 0.2 mm, 3 months- Control: 2.30 ± 0.3 mm, AZI: 2.23 ± 0.3 mm, 6 months-Control: 2.46 ± 0.3 mm, AZI: 2.32 ± 0.4 mm PPD reduction in deep pockets: 1 month- Control: 4.11 ± 0.5 mm, AZI: 4.34 ± 0.9 mm, 3 months- Control: 4.16 ± 0.4 mm, AZI: 4.46 ± 0.8 mm, 6 months-Control: 0.39 ± 0.4 mm, AZI: 4.88 ± 1.1 mm Overall: P < 0.05 for Control and AZI both
Fonseca et al. 2015 [29]	FMD Control: Baseline 2.39 ± 0.99 mm. 3 month: 2.17 ± 0.77 mm, 6 month 2.20 ± 0.74 mm at 180 days AZI: Baseline 2.73 ± 1.15 mm, 3 month: 2.68 ± 1.14 mm, 6 month- 2.61 ± 1.15 mm PMD Control: Baseline: 2.6 ± 1.19 mm. 3 month: 2.46 ± 1.11 mm, 6 month-2.41 ± 1.04 mm AZI: Baseline: 2.38 ± 0.79 mm, 3 month: 2.27 ± 0.71 mm, 6 month- 2.25 ± 0.72 mm P < 0.05 at 3 months for control and AZI	FMD Control group: Baseline 2.27 ± 0.60 mm, 3 month- 2.09 ± 0.52 mm, 6 month- 2.08 ± 0.52 mm AZI: Baseline 2.20 ± 0.41 mm, 3 month- 2.11 ± 0.41 mm, 6 month- 1.93 ± 0.42 mm PMD Control: Baseline: 2.20 ± 0.55 mm, 3 month: 1.98 ± 0.54 mm, 6 month:1.93 ± 0.47 mm AZI: Baseline: 2.31 ± 0.4 mm, 3 month- 2.21 ± 0.40 mm, 6 month- 2.18 ± 0.40 mm
Martande et al. 2016 [31]	Baseline Study Mean: 7.76 mm Control group: Baseline 7.63 ± 1.42 mm; 1 month- 6.80 ± 1.32, 3 month − 6.40 ± 1.06 mm, 6 month 6.06 ± 1.11 mm, 12 month-5.91 ± 1.09 mm AZI: Baseline 7.69 ± 1.02 mm; 1 month- 6.69 ± 1.17, 3 month- 5.54 ± 0.89 mm, 6 month- 5.16 ± 1.10 mm, 12 month- 4.97 ± 1.18 mm Between group difference: 3 months (*P* < 0.0005), 6 months (*P* = 0.0013), 12 months (*P* = 0.0009).	Baseline Study Mean: 6.66 mm Control group; Baseline 6.74 ± 1.40 mm; 1 month- 6.00 ± 1.29, 3 month- 5.57 ± 1.17 mm, 6 month- 5.43 ± 1.24 mm, 12 month- 5.23 ± 0.73 mm. AZI: Baseline 6.57 ± 0.85 mm; 1 month- 5.29 ± 1.05, 3 month- 4.46 ± 0.92 mm, 6 month- 3.74 ± 0.78 mm, 12 month-3.66 ± 0.73 mm. Between group difference: 1 month (*P* = 0.0024), 3 months (*P* < 0.0001), 6 months (P < 0.0001), and 12 months (P < 0.0001)

*AZI* Test group: Azithromycin + S/ RSD Group. *C* Control Group; SRSD alone, *FMD* Full mouth debridement, *PMD* Partial Mouth S/RSD,

(studies are ordered by year of publication)

### Compliance and adverse events

The majority of the studies analyzed compliance by counting the number of tablets provided by the patient. One paper did not report on the outcome of compliance [30] whereas one study reported of a non-compliant participant [28]. Adverse event reporting included diarrhea, reported in 4 studies, which involved 5 subjects (Table 1). One study reported a case of an allergic reaction and also difficulty in swallowing [32]. Another study had subjects complaining of excessive sleepiness and dizziness [34]. Three trials reported no adverse events [29, 31, 33] while one did not document them [30].

### Meta-analyses of primary outcomes

**Fig. 3:**
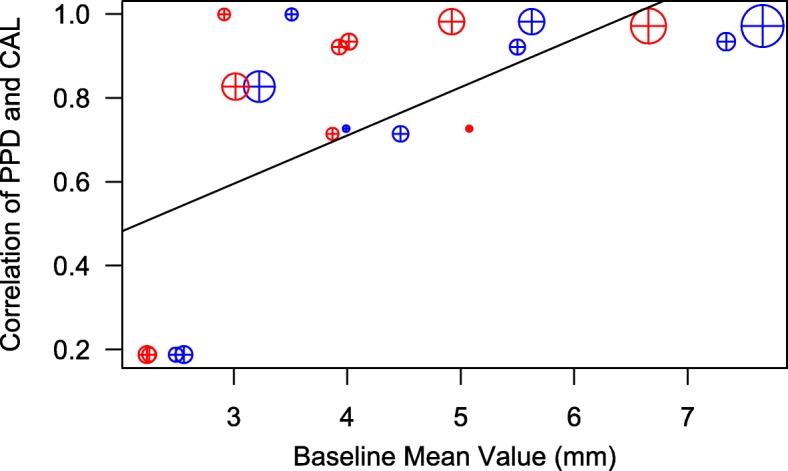
Within-study correlation of primary outcomes (Spearman’s r-value; PPD and CAL, y-axis) plotted against study-level Baseline Mean Values (blue: CAL, red: PPD, x-axis). The size of the circle corresponds to the number of subjects in the study

**Fig. 4:**
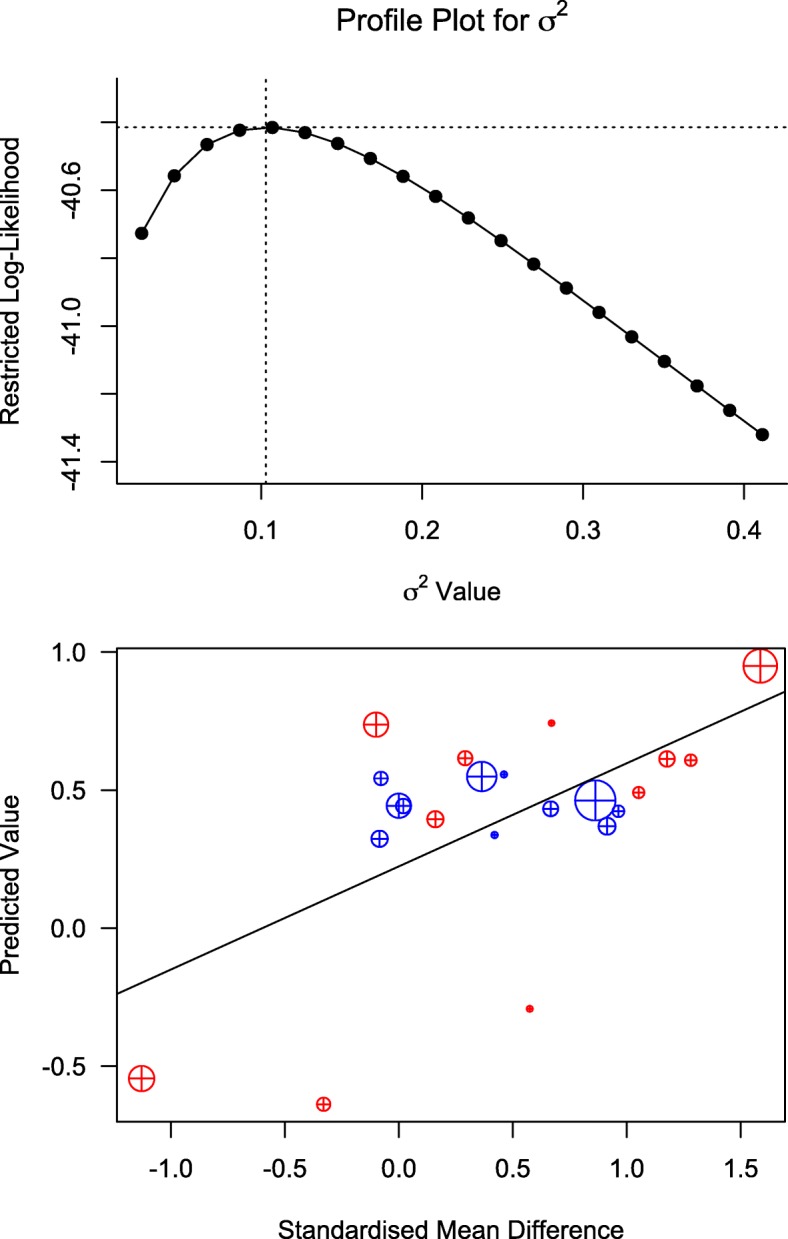
Model performance: 4a: The profile likelihood plot showing study-level random variance component (Sigma squared) for the full-model and 4b: the actual versus the predicted SMD values (blue: CAL, red: PPD, The size of the circle corresponds to the number of subjects in the study)

**Fig. 5:**
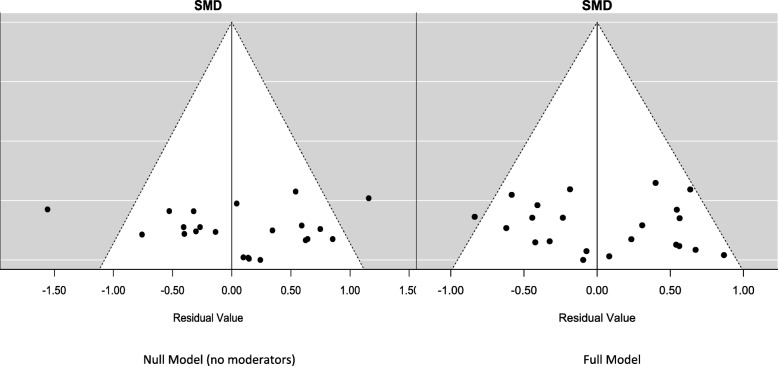
Funnel plots for outcomes for the full-model (left) showed lower asymmetry than the reduced model (right)

**Fig. 6:**
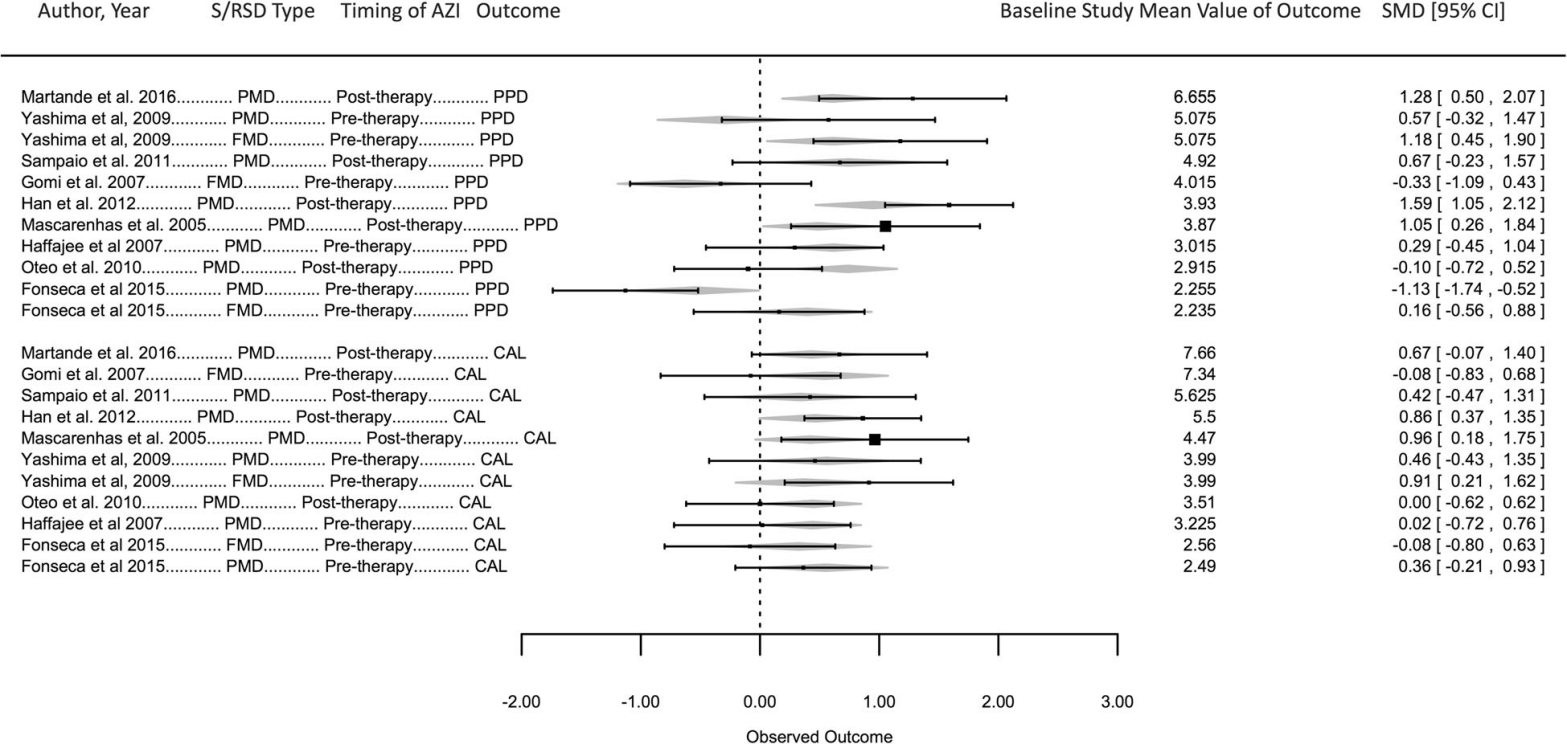
Forest plot depicting the full-model and moderators. The observations are ordered according to Outcome (PPD, CAL) and decreasing Baseline Study Mean Values

**Fig. 7:**
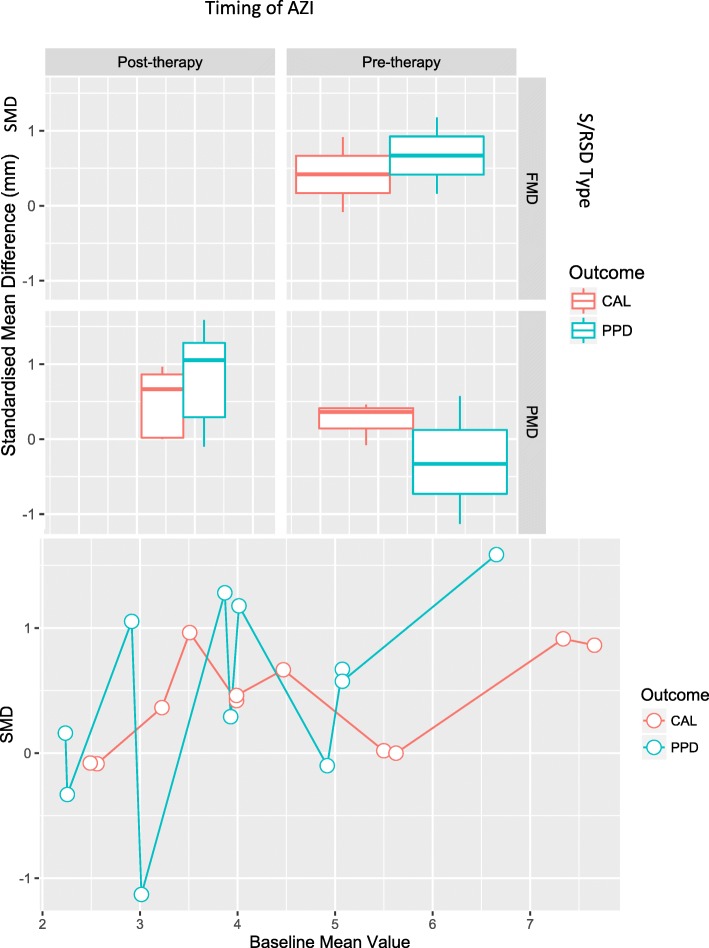
**a**) Bar plots depicting the Standardized mean difference (SMD) in Outcomes within categories of ‘type of S/RSD’ (vertical panels) and ‘timing of drug’ (horizontal panels -top) and **b**) Line plot showing Standardized mean difference (SMD) variation (y-axis) with Baseline mean value of PPD and CAL (x-axis) (bottom)

The meta-analysis was performed for 11 observations from the 9 studies and results are summarized in Table 3. A significant correlation of CAL versus PPD correlation coefficients (range: 0.18–0.73) and their study Baseline mean values (range: 2.2 mm – 6.7 mm for PPD and 2.5–7.7 mm for CAL) was noted (Fig. 3) indicating that CAL and PPD co-vary increasingly as their values increase (Spearman’s correlation cofficient = 0.79, *p* < 0.01). The model performance was satisfactory (Table 3). The full-model’s profile likelihood plot of the study-level random variance component peaked at the parameter estimate (0.103) (Fig. 4a) and predicted versus actual SMD values showed a linear relationship (Fig. 4b), reflecting acceptable model performance. Funnel plots (Fig. 5) showed the reduced model had higher heterogeneity for SMD than the full model, for which the plot closely approximated symmetry. The full model was significantly different and had lower AIC (94.2 versus 185.2) compared to the reduced model (LRT = 102.94 p < 0.01). A forest plot of the full-model is shown in Fig. 6, compared to a forest plot of the no moderators model. In the full model, a significant effect was seen for only on the SMD for PPD (estimated SMD = 1.16, 95% CI: 0.27–2.07, *p* = 0.01). Significant positive interaction was noted for PPD with mean Baseline value (estimated SMD = 0.11, 95% CI: 0.08–0.15) and negative interaction was seen for PPD with PMD (estimated SMD = -1.25, 95% CI = − 1.73- -0.78) and pre-therapy drug initiation (estimated SMD = -1.18, 95% CI = − 1.48- -0.87) (Fig. 7) (Table 3).

**Table 3:** Results of the multivariate meta-analysis

Interacrtions in the full model = Outcome*Baseline Value, Outcome*S/RSD Type, Outcome*Timing					
*Parameter*			*Estimated SMD*	*P value*	*95% C.I*
Outcome: CAL			0.17	0.76	-0.92-1.26
**Outcome: PPD**			**1.17**	**0.01**	0.27–2.07*
Baseline Value			0.01	0.87	−0.10-0.12
S/RSD Type: PMD (reference value = FMD)			0.22	0.50	−0.42-0.86
Timing: Pre-therapy (reference value = Post-Therapy)			0.13	0.72	−0.57-0.83
PPD*Baseline			**0.11**	**< 0.01**	0.08–0.15***
PPD*S/RSD Type PMD			**−1.25**	**< 0.01**	−1.73--0.78***
PPD*Timing: Pre-therapy			**−1.18**	**< 0.01**	−1.48--0.87***
*Model Statistics*					
*Log Likelihood ratio*	*Deviance*	*AIC*	*BIC*		
−38.06	76.13	94.20	99.88		
Variance Components: Study, number of levels = 09, Estimated 휎2 value: 0.10					
*Test for Residual Heterogeneity, Moderators and Model Comparison*					
*Test for Residual Heterogeneity:* **QE (df = 14) = 88.50,** ***p*** **-value < 0.01** *Test of Moderators:* **QM (df = 8) = 150.03, p-value < 0.01** *Model comparsion with null model without moderators (ANOVA)*					
	AIC	Log Likelihood ratio	Likelihood Ratio Test Statistic	P value	Model QE
Full model (df = 9)	94.20	−38.06			88.50
Reduced (df = 3)	185.23	−89.54	103.10	**< 0.01**	201.50

*p < 0.05, **p < 0.01, *** *p* < 0.0001, Significant values in bold font

## Discussion

The current study applied a multivariate approach to jointly synthesize PPD and CAL outcomes in AZI + S/RSD as compared to S/RSD alone in the treatment of chronic periodontitis [15]. Despite high concordance of the included literature with the two previous meta-analyses [13, 14], this approach was distinctive in accommodating varying within-study correlations of the main outcome parameters and exploring possible sources of variability within a single synthesis. As such, its main purpose was to attempt some resolution of the previously noted heterogeneity in clinical outcomes. The major finding was that when PPD and CAL were modeled together factoring in their within-study correlations and between-study variation was accounted for as a random variable, a significant effect of AZI + S/RSD was noted only for PPD. This was a major differentiating point from the results of the two past meta-analysis [13, 14]. Multiple correlated outcome measures, such as key clinical periodontal measures, present a particular challenge in synthesis. As noted previously [15], we found the correlation of PPD and CAL increased with increasing baseline values of these parameters (Fig. 3), which suggested this relationship would significantly impact the outcomes. A multivariate approach resolves this issue by joint synthesis of these outcomes by accounting for the covariance. It has been shown as a valuable approach in synthesizing treatment outcomes of periodontal disease [19], however, not widely applied. The major diagnostic criterion for chronic periodontitis has been the presence of clinical attachment loss. As a result, study populations may be expected to vary in probing pocket depth distribution, as varying amounts of clinical attachment loss may present as recession. In agreement, the study mean values for these outcomes ranged 2.2 mm – 6.7 mm for PPD and 2.5–7.7 mm for CAL (Table 1), reflecting a wide range of clinical disease severities and within-study correlations of PPD and CAL (Spearman’s r = 0.19–0.73).

Notably, the confidence interval for the summary PPD outcome was very large (0.27–2.06 mm) again suggesting the actual clinical benefit is variable, possibly depending on background factors. Two issues are relevant to this finding. Firstly, the clinical measurement of probing depth and clinical attachment loss with manual probing is inherently subject to measurement error. The magnitude of such error has classically been reported as 0.36 mm and 0.41 mm respectively [35]. Moreover, in untreated periodontitis, where there is lower resistance to probing and presence of bleeding and root surface calculus, measurement variability in manual probing may be relatively higher [36]. Taken together, it appears that the 95% CI for SMD accorded to the benefit of adjunctive AZI overlaps with the documented range of clinical error in probing, which may be a source of potential bias. A second issue is the translation of the additional reduction in PPD due to adjunctive AZI to a reduction in the need for future surgery. A previous synthesis, which assessed the value of adjunctive Amoxicillin and Metronidazole to S/RSD found that despite a clinical benefit in PPD reduction at 3–6 months, no notable benefit in terms of a need for periodontal surgery, as indicated by a PPD of 3 mm or less, was evident [37]. These authors also noted high heterogeneity in the included studies, similar to the present synthesis. While we did not directly extrapolate the need for periodontal surgery as an outcome, the large range of the 95% CI in PPD reduction appears cautionary against a blanket assumption of such benefit attributable to adjunctive AZI in all chronic periodontitis-affected sites. This finding also emphasizes the need to investigate the sources of variability in clinical outcomes as these would point to patient profiles who may stand to benefit from this intervention.

Although significant residual heterogeneity remained in the full model, the test for moderators was significant and it explained the outcomes significantly better as compared to a model without any moderators. The moderator interaction effects were addressed in a single synthesis, unlike past meta-analysis [14]. Two of the 3 included moderators (type of S/RSD and timing of drug) have not been addressed in the context of adjunctive AZI outcomes in S/RSD. For every 1 mm increase in Baseline PPD, the estimated SMD for AZI + S/RSD increased significantly by 0.11 mm. Thus, increasingly deeper pockets thus benefited the most from adjunctive systemic AZI at 6-months post-therapy, in agreement with a previous meta-analysis [14]. We did not categorize the pockets into deep, moderate, and shallow, as a uniform categorization across all the included primary literature was lacking. Considering Baseline study mean value as a moderator allowed to test its impact as a continuous variable applicable to all the included cases. Arguably, this approach provided for better statistical robustness as compared to the analysis of a subset of studies alone which grouped pockets based on depth-cut offs. In theory, greater benefit in PPD reduction of increasingly deeper sites may translate to the lower need for surgical intervention or lower risk of disease progression when AZI + S/RSD is applied in deep probing sites. However, a cut-off value of Baseline PPD where this approach lowers the need for periodontal surgery or prevents further loss of attachment is unclear. These questions should be addressed by well-designed clinical trials focussed on these two particular outcomes, with stringent inclusion criteria.

A PMD approach showed an estimated significantly lower SMD in PPD by 1.25 mm over the FMD approach and pre-therapy drug initiation resulted in an estimated lower SMD 1.18 mm as compared to post-therapy drug initiation. These effect sizes are comparable to the overall main effect for PPD (1.17 mm) but with smaller confidence intervals (Table 3). The finding that FMD was superior to PMD is largely in agreement with the full-mouth disinfection concept, which advocates a conjunctive application of an antimicrobial agent with complete mechanical debridement [20]. An FMD approach to S/RSD itself has not shown a clearly increased benefit [38]. Notably, we did not consider the number of sittings and their intervals over which PMD was performed. These were variable across studies and may be relevant factors to consider when comparing PMD with the FMD approach. The drug half-life for AZI is 7 days. With this basis, Yashima et al. (2009) [27] hypothesized that when PMD is delivered within this time-frame, clinical benefits would be comparable to the FMD approach as compared to the typical PMD delivery in a quadrant wise approach provided over 2–4 weekly intervals, as noted in 7 of the included studies. This typical approach would result in the debridement of some areas being performed after the effective concentration of the drug declined to non-therapeutic levels, which may be responsible for a lower benefit.

Much less is known about any impact of the time-point of drug initiation. From a biological standpoint, the time of drug initiation may lead to a difference in drug therapeutic concentrations achieved either before or after the disruption of the periodontitis-related biofilm. Secondly, it may affect whether the drug is locally active in a state of greater gingival crevicular fluid flow and pocket-epithelial disruption induced by mechanical instrumentation. Particularly, as AZI tends to sequestrate in the gingival crevicular fluid (GCF) more than in the serum [39] and peak GCF concentration occurs at 36 h [40], it seems plausible that its pharmacokinetics may be used to an advantage by an immediate post-S/RSD drug regime. This would be particularly relevant to the PMD approach when the sittings are widely spaced. Initiation in conjunction with or earlier to S/RSD would result in high drug concentrations where intact subgingival biofilm still remains which may result in a lower clinical benefit, as noted in pre-therapy initiation in this study. In an FMD approach, it is conceivable that starting the drug prior to or immediately after S/RSD would not impact its efficacy much as drug half-life would coincide with the post-debridement time-frame. The potential interaction between drug timing and S/RSD type was not examined as there were no cases where an FMD approach was used with post-therapeutic intervention.

Taken together, the multivariate approach resolved part of the heterogeneity noted in clinical outcomes of adjunctive AZI in S/RSD in the treatment of chronic periodontitis. Study-level differences in drug timing, baseline disease severity, and type of S/RSD appear to impact the degree of reduction in PPD attributed to adjunctive AZI at 6 months after S/RSD. The positive study-level random variance (sigma 2) component depicted the existence of between-study heterogeneity. Persistent residual heterogeneity was noted in the full-model suggesting many unexamined potential moderators exist. Chief among these would be smoking, which reduces healing response to S/RSD [41], plaque control and supportive therapy, which are key determinants of outcome of nonsurgical periodontal therapy [42, 43]. Notably, the maintenance regimes followed by the studies included varied considerably in terms of the time interval. The major limitation of this synthesis was a small number of qualifiable studies for analyses. Large-scale, standardized and well-designed RCTs are required to investigate the moderators suggested by this analysis and facilitate concrete recommendations. Clinical end-points in future studies should include the proportion of remaining deep pockets, sites that undergo progression, need for surgery, and patient-related outcomes.

## Conclusion

Adjunctive systemic AZI with S/RSD can confer significant additional but variable clinical benefit in PPD reduction at 6 months after therapy as compared to scaling and root surface debridement alone. The magnitude of this benefit is influenced by baseline disease severity, the timing of the drug initiation, and type of non-surgical therapy. Single sitting full-mouth debridement and drug initiation post-instrumentation have a beneficial interaction with this intervention.

## Additional file

Additional file 1: **Table S1.** Description of Pubmed Search. **Table S2.** Excluded studies with reasons for exclusion. **Table S3.** Description of Clinical Measures (CAL, PPD, BOP) reported in the included studies. **Table S4.** Assessment of Publication Bias Assessments (Begg’s and Egger’s regression test *p* values). (DOCX 24 kb)

## Abbreviations

AZI: Azithromycin
BOP: Bleeding on probing
CAL: Clinical attachment level
FMD: Full-mouth debridement
PMD: Partial-mouth debridement
PPD: Probing depth
S/RSD: Scaling and root surface debridement
SMD: Standardised mean difference
