# Supplementary material for: Disease severity, debridement approach and timing of drug modify outcomes of adjunctive azithromycin in non-surgical management of chronic periodontitis: a multivariate meta-analysis
Source: BMC Oral Health. 2019 Apr 27;19:65. doi: 10.1186/s12903-019-0754-0 (PMC6486979; doi:10.1186/s12903-019-0754-0)
Supplement: Supplementary file 1 — Table S1. Description of Pubmed Search. Table S2. Excluded studies with reasons for exclusion. Table S3. Description of Clinical Measures (CAL, PPD, BOP) reported in the included studies. Table S4. Assessment of Publication Bias Assessments (Begg’s and Egger’s regression test p values). (DOCX 24 kb) [file 12903_2019_754_MOESM1_ESM.docx]

**Table S1: Pubmed Search Terms**

| #1 | Azithromycin [MeSH Terms] |
| --- | --- |
| #2 | Periodontitis [MeSH Terms] or Periodontal Disease [MeSH Terms] |
| #3 | Periodontal Treatment or Periodontal Therapy |
| #4 | #1 AND #2 |
| #5 | #1 AND #3 |
| #6 | #4 OR #5 |

**Table S2: Excluded studies and reasons for exclusion**

| **Author and Year** | **Reasons for exclusion** |
| --- | --- |
| Nepokupnaia et al. 2014 [66] | Publication in Georgian Language |
| Nepokupnaia et al. 2014 [67] | Publication in Russian Language |
| Schmidt et al. 2011 [68] | Not a RCT, Surgical Treatment during follow-up |
| Ali et al. 2011 [69] | Poster presentation |
| Schmidt et al. 2007 [70] | Case report |
| Shibukawa et al. 2003 [71] | Publication in Japanese Language |
| Smith et al. 2002 [72] | Follow-up only for 3 months |
| Tsarev et al. 1997 [73] | Publication in Russian Language |
| Filatova et al. 1995 [74] | Publication in Russian Language |
| Jentsch, 2016 [75] | 6 month outcomes were not reported |
| Miremadi, 2015 [50] | Azithromycin was provided during maintenance |
| Nakajima, 2016 [76] | No control group with S/RSD alone |
| Saleh, 2016 [77] | Outcomes were reported at 3 months |

**Table S3: Clinical Measures (CAL, PPD, BOP)**

| **Study**  **CAL** | **Baseline** | **AZI+ S/RSD** | | **S/RSD** |  | **Timing of AZI** | **Type of S/RSD** |
| --- | --- | --- | --- | --- | --- | --- | --- |
|  |  | Mean (SD) | N | Mean (SD) | N |  |  |
| Mascarenhas et al. (2005) | 4.47 | 1.13 (1.16) | 15 | 0.46 (0.75) | 15 | Post-therapy | Qu |
| Gomi et al. (2007) | 7.34 | 2.62(1.44) | 17 | 1.47 (0.98) | 17 | Pre-therapy | FMD |
| Oteo et al. (2010) | 3.51 | 0.76 (0.46) | 15 | 0.28 (0.50) | 13 | Post-therapy | Qu |
| Sampaio et al. (2011) | 5.625 | 1.05 (1.56) | 20 | 1.05 (1.54) | 20 | Post-therapy | Qu |
| Han et al. (2012) | 5.5 | 1.55 (0.5) | 14 | 1.54 (0.5) | 14 | Post-therapy | Qu |
| Martande et al. (2016) | 7.66 | 2.51 (0.78) | 35 | 1.57 (1.31) | 35 | Post-therapy | Qu |
| Haffajee et al. (2007) | 3.225 | 0.17 (0.12) | 25 | 0.12 (0.15) | 23 | Pre-therapy | Qu |
| Yashima et al. (2009)a | 3.99 | 1.18 (0.46) | 10 | 0.96 (0.54) | 10 | Pre-therapy | FMD |
| Yashima et al. (2009)b | 3.99 | 1.24 (0.62) | 10 | 0.96 (0.54) | 10 | Pre-therapy | Qu |
| Fonseca et al. (2015)a | 2.56 | 0.12 (0.89) | 15 | 0.19 (0.71) | 15 | Pre-therapy | FMD |
| Fonseca et al. (2015)b | 2.49 | 0.13 (0.59) | 14 | 0.19 (0.87) | 13 | Pre-therapy | Qu |
| Total |  |  | 190 |  | 185 |  |  |
| **Study**  **PPD** | **Baseline** | **AZI+ S/RSD** | | **S/RSD** |  | **Timing of AZI** | **Type of S/RSD** |
|  |  | Mean (SD) | N | Mean (SD) | N |  |  |
| Mascarenhas et al. (2005) | 3.87 | 1.33 (0.73) | 15 | 0.45 (0.60) | 15 | Post-therapy | Qu |
| Gomi et al. (2007) | 4.02 | 1.62 (0.86) | 17 | 0.75 (0.55) | 17 | Pre-therapy | FMD |
| Oteo et al. (2010) | 2.92 | 0.78 (0.34) | 15 | 0.38 (0.40) | 13 | Post-therapy | Qu |
| Sampaio et al. (2011) | 4.92 | 1.54 (1.62) | 20 | 1.71 (1.71) | 20 | Post-therapy | Qu |
| Han et al. (2012) | 3.93 | 1.81 (0.5) | 14 | 1.66 (0.50) | 14 | Post-therapy | Qu |
| Martande et al. (2016) | 6.66 | 2.82 (0.89) | 35 | 1.31 (0.99) | 35 | Post-therapy | Qu |
| Haffajee et al. (2007) | 3.02 | 0.3 (0.11) | 25 | 0.45 (0.15) | 23 | Pre-therapy | Qu |
| Yashima et al. (2009)a | 5.08 | 2.17 (0.79) | 10 | 1.52 (1.05) | 10 | Pre-therapy | FMD |
| Yashima et al. (2009)b | 5.08 | 2.06 (0.72) | 10 | 1.52 (1.05) | 10 | Pre-therapy | Qu |
| Fonseca et al. (2015)a | 2.56 | 0.12 (0.89) | 15 | 0.19 (0.71) | 15 | Pre-therapy | FMD |
| Fonseca et al. (2015)b | 2.49 | 0.13 (0.59) | 14 | 0.19 (0.87) | 13 | Pre-therapy | Qu |
| Total |  |  | 190 |  | 185 |  |  |
| **Study**  **BOP** | **Baseline** | **AZI+ S/RSD** | | **S/RSD** |  | **Timing of AZI** | **Type of S/RSD** |
|  |  | Mean (SD) | N | Mean (SD) | N |  |  |
| Mascarenhas et al. (2005) | 63.29 | 18.6 (35.32) | 15 | 17.71(23.21) | 15 | Post-therapy | Qu |
| Gomi et al. (2007) | 31.43 | 26.07 (16.81) | 17 | 18.52(18.51) | 17 | Pre-therapy | FMD |
| Oteo et al. (2010) | 46.57 | 31.27 (15.14) | 15 | 24.55(16.80) | 13 | Post-therapy | Qu |
| Sampaio et al. (2011) | 78.86 | 65.87 (24.13) | 20 | 69.62(14.07) | 20 | Post-therapy | Qu |
| Han et al. (2012) | 68.00 | 54.08 (19.30) | 14 | 50.29(17.20) | 14 | Post-therapy | Qu |
| Martande et al. (2016) | 71.03 | 52.65 (9.28) | 35 | 47.31(9.91) | 35 | Post-therapy | Qu |
| Haffajee et al. (2007) | 33.46 | 24.00 (3.90) | 25 | 19.5(3.90) | 23 | Pre-therapy | Qu |
| Yashima et al. (2009)a | 44.05 | 36.74 (14.85) | 10 | 33(13.62) | 10 | Pre-therapy | FMD |
| Yashima et al. (2009)b | 44.05 | 36.74(14.85) | 10 | 33(13.62) | 10 | Pre-therapy | Qu |
| Total |  |  | 161 |  | 157 |  |  |

S/RSD – Scaling and Root Surface Debridement, AZI - Azithromycin, Qu - Quadrant wise S/RSD, FMD- Full-mouth debridement

**Table S4: Publication Bias Assessments**

| **Outcome** | **Begg’s**  **Regression**  **P value** | **Egger Regression**  **P value** |
| --- | --- | --- |
|  |  |  |
| **CAL**  **Gain** | 0.64 | 0.06 |
| **PPD**  **Reduction** | 0.44 | 0.01 |
| **BOP**  **Changes** | 0.12 | 0.60 |
